# Construction and Validation of a Tumor Microenvironment-Based Scoring System to Evaluate Prognosis and Response to Immune Checkpoint Inhibitor Therapy in Lung Adenocarcinoma Patients

**DOI:** 10.3390/genes13060951

**Published:** 2022-05-26

**Authors:** Pinzheng Huang, Linfeng Xu, Mingming Jin, Lixi Li, Yizhong Ke, Min Zhang, Kairui Zhang, Kongyao Lu, Gang Huang

**Affiliations:** 1 School of Health Science and Engineering, University of Shanghai for Science and Technology, Shanghai 200093, China; 193832401@st.usst.edu.cn (P.H.); linfneg@163.com (L.X.); 193832330@st.usst.edu.cn (L.L.); 192672192@st.usst.edu.cn (Y.K.); 193832310@st.usst.edu.cn (M.Z.); 192672174@st.usst.edu.cn (K.Z.); 192672195@st.usst.edu.cn (K.L.); 2 Shanghai Key Laboratory of Molecular Imaging, Shanghai University of Medicine and Health Sciences, Shanghai 201318, China; asdjinmingming@126.com; 3 Shanghai Institute for Biomedical and Pharmaceutical Technologies, Shanghai 200030, China

**Keywords:** lung adenocarcinoma, tumor microenvironment, prognostic stratification, immune checkpoint inhibitor therapeutic response evaluation, TIDE score

## Abstract

Background: Lung cancer is among the most dangerous malignant tumors to human health. Lung adenocarcinoma (LUAD) accounts for about 40% of all lung cancers. Accumulating evidence suggests that the tumor microenvironment (TME) is a crucial regulator of carcinogenesis and therapeutic efficacy in LUAD. However, the impact of tumor microenvironment-related signatures (TMERSs) representing the TME characteristics on the prognosis and therapeutic outcome of LUAD patients remains to be further explored. Materials and methods: Gene expression files and clinical information of 1630 LUAD samples and 275 samples with immunotherapy information from different databases such as The Cancer Genome Atlas (TCGA), Gene Expression Omnibus (GEO) and Cancer Research Institute (CRI) iAtlas were downloaded and analyzed. Three hundred tumor microenvironment-related signatures (TMERS) based on a comprehensive collection of marker genes were quantified by single sample gene set enrichment analysis (ssGSEA), and then eight significant signatures were selected to construct the tumor microenvironment-related signature score (TMERSscore) by performing Least Absolute Shrinkage and Selection Operator (LASSO)-Cox analysis. Results: In this study, we constructed a TME-based prognostic stratification model for patients with LUAD and validated it in several external datasets. Furthermore, the TMERSscore was found to be positively correlated with tumor malignancy and a high TMERSscore predicted a poor prognosis. Moreover, the TMERSscore of responders treated with Immune Checkpoint Inhibitor (ICI) therapies was significantly lower than that of non-responders, and the TMERSscore was positively correlated with the tumor immune dysfunction and exclusion (TIDE) score, implying that a low TMERSscore predicts a better response to ICI treatment and may provide independent and incremental predictive value over current biomarkers. Conclusions: Overall, we constructed a TMERSscore that can be used for LUAD patient prognosis stratification as well as ICI therapeutic efficacy evaluation, supportive results from independent external validation sets showed its robustness and effectiveness.

## 1. Introduction

Lung cancer is now the leading cause of cancer-related death worldwide [1]. Non-small cell lung cancer (NSCLC) accounts for 85% of lung cancers and lung adenocarcinoma (LUAD) is the most common type of NSCLC, accounting for about 40% of all lung cancer cases [2]. Despite recent advances in surgery, chemotherapy, radiotherapy, targeted therapy, and immunotherapy, 5-year survival rates for patients with LUAD remains poor [3,4]. In order to improve patient prognosis and develop individualized treatment, strong and robust differentiation criteria are urgently needed to guide patient prognostic stratification. Although many studies have proposed biomarkers or gene signatures that may predict the prognosis of LUAD, most of them are based only on single or multiple gene combinations, do not explore the common role of gene sets from a systems biology perspective, and are still not applied in clinical practice. Therefore, the discovery of new gene-set-based prognostic biomarkers for LUAD will be of great importance.

The tumor microenvironment (TME) refers to the surrounding tumor environment including peripheral blood vessels, immune cells, fibroblasts, signaling molecules, and extracellular matrix [5,6]. Tumors are closely related to, and interact with, the surrounding microenvironment; they can also affect the microenvironment by releasing extracellular signals, promoting tumor angiogenesis and inducing peripheral immune tolerance, while immune cells in the microenvironment can influence the growth and evolution of cancer cells [7,8]. Signaling molecules such as HLA [9], IFN response [10], cytokines [11], inflammatory factors and immune checkpoints [12] in the tumor microenvironment have proven to play an important role in the evolution of lung cancer. Many biomarkers for assessing prognosis and therapeutic efficacy from TME have been shown to be effective in lung cancer [13,14,15].

Immune Checkpoint Inhibitor (ICI) therapies based on TME have made many achievements, such as programmed cell death protein 1 (PD-1)/programmed death-ligand 1 (PD-L1) and CTLA4 checkpoint inhibitors, which have revolutionized the treatment of NSCLC in the last decade [16,17]. Immunotherapy using ICIs is now the standard of care for the treatment of lung cancer, breast cancer and other solid tumor types [18,19]. Although ICIs can improve clinical outcomes in patients with a diversity of solid tumors, the majority of unselected patients do not respond [20,21]. In addition, ICIs may cause immune-related adverse events, some of which are clinically serious to potentially life-threatening [22,23]. Therefore, the establishment of biomarkers that can identify patients who are more likely to benefit from ICI therapy is essential to optimize the use of immunotherapy.

In this study, based on a comprehensive collection of marker genes attached to TME-related signatures (TMERSs) in the literature, multiple datasets from different sources were used to obtain the quantitative results of these TMERSs by single sample gene set enrichment analysis (ssGSEA), where each TMERS is a gene set and ssGSEA calculates an enrichment score for each TMERS based on the expression level of the genes contained in each TMERS. Then, eight significant signatures were selected based on the LASSO-Cox analysis to construct a tumor microenvironment-related signature score (TMERSscore). The TMERSscore was successfully validated for prognosis stratification of LUAD patients in multiple LUAD datasets by comparing normal versus disease samples, and early versus advanced-staging samples. In addition, TMERSscore was positively correlated with the TIDE score, and in the NSCLC and skin cutaneous melanoma (SKCM) patients treated with ICIs, the TMERSscores were significantly lower in responders than in non-responders, implying that the TMERSscore is a potential biomarker that can be used to evaluate the prognosis and the response to ICI therapy for patients with LUAD.

## 2. Materials and Methods

### 2.1. Data Acquisition and Preprocessing

In this study, we downloaded the gene expression profiles and corresponding clinical information of LUAD patients from the Cancer Genome Atlas (TCGA) database as training data, and samples without sufficient clinical information were excluded (e.g., follow-up time, follow-up status, AJCC pTNM stage, T stage, N stage), and only two samples belonging to N3 stage in the project were also excluded. The probe expression files of GSE30219, GSE31210, GSE68465, GSE72094 and the corresponding clinical information were downloaded from the Gene Expression Omnibus (GEO) database as the validation data for survival prediction, only the LUAD samples with complete survival information were applied in these datasets, and information about all samples included in the construction and validation of the survival prediction model is shown in Table 1. LUAD samples from the TCGA-LUAD project and GSE32863 and their matched adjacent non-tumor lung tissue were used to assess the difference in TMERSscore between paired normal and tumor samples. NSCLC samples from GSE126044 receiving anti-PD-1 therapy and SKCM samples from the Cancer Research Institute (CRI) iAtlas [24] platform receiving ICI therapies were also utilized to evaluate the performance of TMERSscore in predicting the response to ICI therapy.

Log2-transformed read counts expression values were obtained for data from TCGA and CRI iAtlas. Then log2-transformed probe expression values were applied in the GEO database and, if a gene matched multiple probe annotations, the expression value of the gene was the average of the expression value of these probes. To enable comparisons across platforms, we performed batch correction of the included datasets by employing the normalization method in the limma package in R [25].

### 2.2. Collection and Quantification of Tumor Microenvironment-Related Signatures

The collection of tumor microenvironment-related features (TMERS) was divided into two main sections based on an extensive literature search. The first portion was the immune cell signatures based on an extensive collection of marker genes mentioned in a literature [26], with a total of 184; the collection of these signatures was derived from different resources. Of them, 25 were derived from the work of Bindea et al. [27], 68 from the study of Wolf et al. [28], 17 were downloaded from the ImmPort database [29], 24 from the study of Miao et al. [30], and another 22, 10 and 10 from CIBERSORT [31], MCPcounter [32] (version 1.2.0), and imsig [33] (version 1.1.3), respectively. The second portion contained 119 TME associated signatures obtained from the R package IOBR [34] (version 0.99.9). The two sections added up to a total of 303 TMERSs, the list of the 303 TMERSs and their contained genes can be found in Supplementary Table S1.

The normalized quantitative enrichment results for each TMERS in each sample were acquired by the ssGSEA method in the GSVA package [35] (version 1.38.2) based on the Limma-corrected gene expression profiles that provides an enrichment score based on the expression level of the genes contained in each TMERS, which represents the absolute enrichment level of each TMERS in each sample. Only 300 TMERSs were evaluated and used for follow-up studies because some marker genes were missing from the corrected gene expression profiles.

### 2.3. Establishment of the Tumor Microenvironment-Related Signature Score

Since some TMERSs with small variance lead to hazard ratio non-convergence, and hazard ratio can be adjusted by amplifying the variance of TMERSs, we amplified all TMERSs by a factor of 100 to increase the variance and use it for subsequent analysis [36]. Based on the quantitative enrichment matrix of LUAD patients in TCGA derived from the above calculation, we first applied the univariate Cox proportional hazards regression analysis with overall survival (OS) as the target, and 18 TMERSs with *p* < 0.001 were selected. Next we employed LASSO regression analysis to further reduce the features and, finally, eight TMERSs, i.e., Bcell_receptors_score, IgG_19272155, T_cells_MCPcounter, B_lineage_MCPcounter, Nucleotide_excision_repair, iDC_Bindea_et_al, SW480_cancer_cells_Bindea_et_al, MDSC_Peng_et_al were obtained and used to construct the TMERSscore.

Multivariate Cox proportional hazards regression analysis was then performed based on the selected eight TMERSs, and the TMERSscore was constructed based on the quantitative enrichment matrix of selected TMERSs and the corresponding regression coefficients as follows:

$$\mathrm{TMERSscore}=\overset{8}{\underset{i=1}{\sum }}\beta_{i}*{\mathrm{TMERS}}_{i}$$

where ${\mathrm{TMERS}}_{i}$ denotes the *i*th TMERS and $\beta_{i}$ denotes the coefficient of multivariate Cox regression analysis of ${\mathrm{TMERS}}_{i}$. In this study, TMERSscores for all samples were calculated by the above formula, and the division between high and low-risk groups in each dataset was based on the optimal cut-off value obtained from the time-dependent ROC curve of the TCGA training cohort for three years, which was obtained using the survivalROC R package (version 1.0.3).

### 2.4. Comparison of Tumor Microenvironment-Related Signature Score with Other Prognostic Models

Three published models for predicting the prognosis of LUAD patients [37,38,39] were used to compare with our model in the TCGA-LAUD cohort, with risk scores for each model calculated by formulas defined in the literature, whose (concordance index) C-index and *p*-values were calculated by the “rcorrcens” function.

### 2.5. Identification of Differentially Expressed Genes

According to the list of 303 TMERSs and their contained genes, we extracted a total of 223 unique genes contained in the selected TMERSs. Then, based on the R package limma and the log2-transformed read counts gene expression profiles in TCGA-LUAD project, those genes with differential abundance between different groups were identified using |log fold change| < 1 and *p*-value < 0.05 as the screening criteria for differential expressed genes (DEGs) [25].

### 2.6. Bioinformatics Analysis of Differentially Expressed Genes

After obtaining a total of 905 DEGs shared between the normal versus disease and low- versus high-risk groups, 34 of the 223 genes contained in the selected TMERSs were found to be differentially expressed in both grouping forms. Then, gene ontology (GO) and Kyoto Encyclopedia of Genes and Genomes (KEGG) enrichment analysis of the 34 genes was performed using the R package clusterProfiler [40] (version 4.1.4). Furthermore, to clarify the key processes activated in the low- and high-risk groups, Gene Set Enrichment Analysis (GSEA) was implemented by utilizing the R package fgsea (version 1.16.0), the classic hallmark gene set (h.all.v7.4.symbols.gmt) from Molecular Signatures Database [41] was considered with adjust *p*-value < 0.05, |NES| > 1 as the reference value.

### 2.7. Evaluation of ICI Treatment Response by Tumor Microenvironment-Related Signature Score

To examine the potency of TMERSscore as a biomarker for predicting the efficacy of ICI treatment in LUAD patients, we calculated the TMERSscores of 16 NSCLC samples receiving anti-PD1 therapy in the GSE126044 dataset and 259 SKCM samples receiving ICI therapy from the CRI iAtlas; gene expression and clinical data for patients in the SKCM cohort are detailed in Supplementary Tables S2 and S3. By comparing TMERSscores between responders and non-responders and between different responses of immunotherapy, we found that non-responders had significantly higher TMERSscores than responders after immunotherapy.

Tumor mutation burden (TMB), which reflects tumor antigenicity and predicts improved survival after immunotherapy, and TIDE score [42], which reflects tumor immune dysfunction and immune escape, are well recognized to assess the benefit of immunotherapy in NSCLC patients, so we obtained the TMB corresponding to LUAD samples in the TCGA cohort based on the R package TCGAmutaions (version 0.3.0) and calculated the TIDE score for these samples. Thereafter, we investigated the correlation between TMERSscore and TMB as well as TIDE score, where low TIDE scores and high TMB were considered to represent a better response to immunotherapy.

### 2.8. Statistical Analysis

Kaplan-Meier survival curves and forest plot showing multivariate Cox regression results were generated by using the R package survminer (version 0.4.9), and log-rank test was used to determine the significance of differences between different groups. Student’s *t*-test was used to compare differences in attribute values between groups. The LASSO algorithm for further feature selection is implemented by the R package glmnet (version 4.1-2). The DCA curve used to assess the benefit of clinical decisions for various risk factors was completed by the R package ggDCA (version 1.2). Univariate and multivariate Cox proportional hazard regression analyses were conducted using survival R package (version 3.2-13), the sensitivity and specificity of TMERSscore were assessed using receiver operating characteristics (ROC), and the visualization of the ROC curves were implemented by the R package timeROC (version 0.4). Boxplots were visualized by using the R package ggpubr (version 0.4.0), *p*-values less than 0.05 were considered to be statistically significant. All statistical analyses were implemented in R software (version 4.0.4).

## 3. Results

### 3.1. Construction of a Tumor Microenvironment-Related Signature Score for Significant Stratification of LUAD Patients

**Figure 1:**
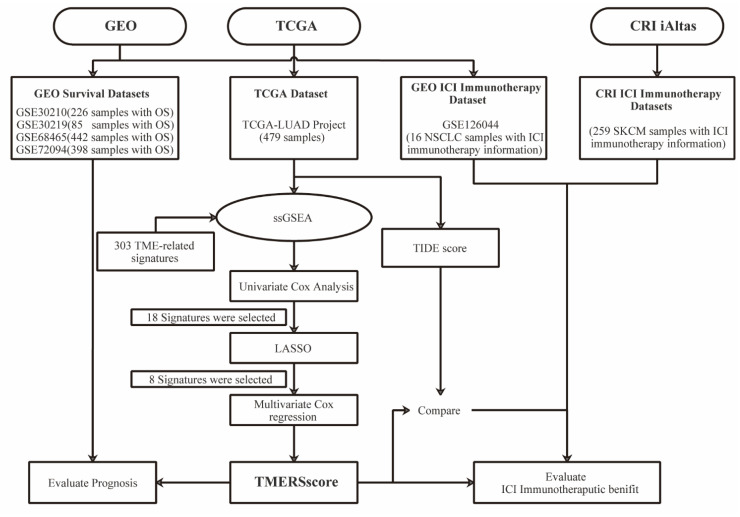
Flowchart of our research process.

**Figure 2:**
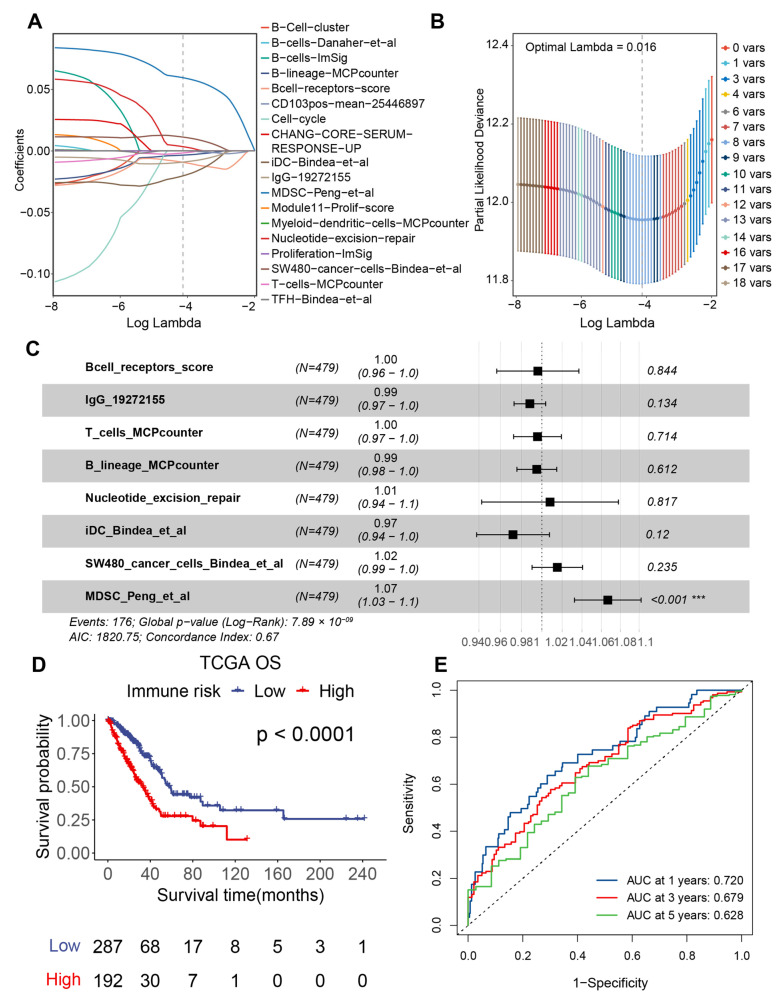
Establishment of TMERSscore. (**A**) LASSO coefficient profiles of the 18 selected TMERSs. (**B**) Adjusting LASSO penalty parameters using 10-fold cross-validation. (**C**) Multivariate Cox regression analysis of the eight selected TMERSs for OS. (**D**) Kaplan-Meier survival curve of the TMERSscore in the training cohort. (**E**) ROC curves of TMERSscore predicting OS. *** *p* < 0.001.

Figure 1 is a flow chart showing the process of our study. Here, 479 LUAD samples with complete clinical information from the TCGA-LUAD project were used as the training set for the prognostic model. Based on the ssGSEA quantitative enrichment matrix of these samples, univariate Cox regression analysis was first used to reveal the relationship between 300 TMERSs and the OS of patients, as shown in the Supplementary Table S4; 18 TMERSs with *p*-values less than 0.001 were selected and used for subsequent analyses. Model feature selection was then further optimized by LASSO regression. After 10-fold cross-validation, LASSO gave the optimal lambda value of 0.016, representing eight variables that were ultimately selected (Figure 2A,B). Subsequently we applied the eight selected TMERSs to the multivariate Cox regression model to construct the TMERSscore (Figure 2C), which is a linear combination of the product of the values of these signatures and their multivariate Cox regression coefficients; the C-index of TMERSscore was 0.67.

All patients in the training set were assigned to the high-risk group (TMERSscore > cut-off value) or the low-risk group (TMERSscore < cut-off value) based on the optimised cut-off value (1.127425) obtained from the TCGA cohort. As depicted in Figure 2D, OS was significantly worse in patients with high TMERSscore than in patients with low TMERSscore (log-rank *p* < 0.0001). To assess the specificity and sensitivity of TMERSscore for predicting OS, a time-dependent ROC analysis was performed using the TMERSscore as a continuous variable. The AUCs of the 1-, 3-, and 5-year ROC curves reached 0.720, 0.679, and 0.628, respectively (Figure 2E), indicating a good performance of the TMERSscore model in the training cohort.

To facilitate a more clinical application we built a nomogram based on the TMERSscore in the TCGA-LUAD cohort that combines the TMERSscore and other risk factors beneficial for clinical decision making (Supplementary Figure S1A). Based on the nomogram, the 1, 3, and 5-year survival rates of patients can be inferred by calculating the sum of the corresponding values. The calibration curve of the nomogram is shown in Supplementary Figure S1B.

### 3.2. Validation of Tumor Microenvironment-Related Signature Score in Predicting Prognosis of LUAD Patients

**Figure 3:**
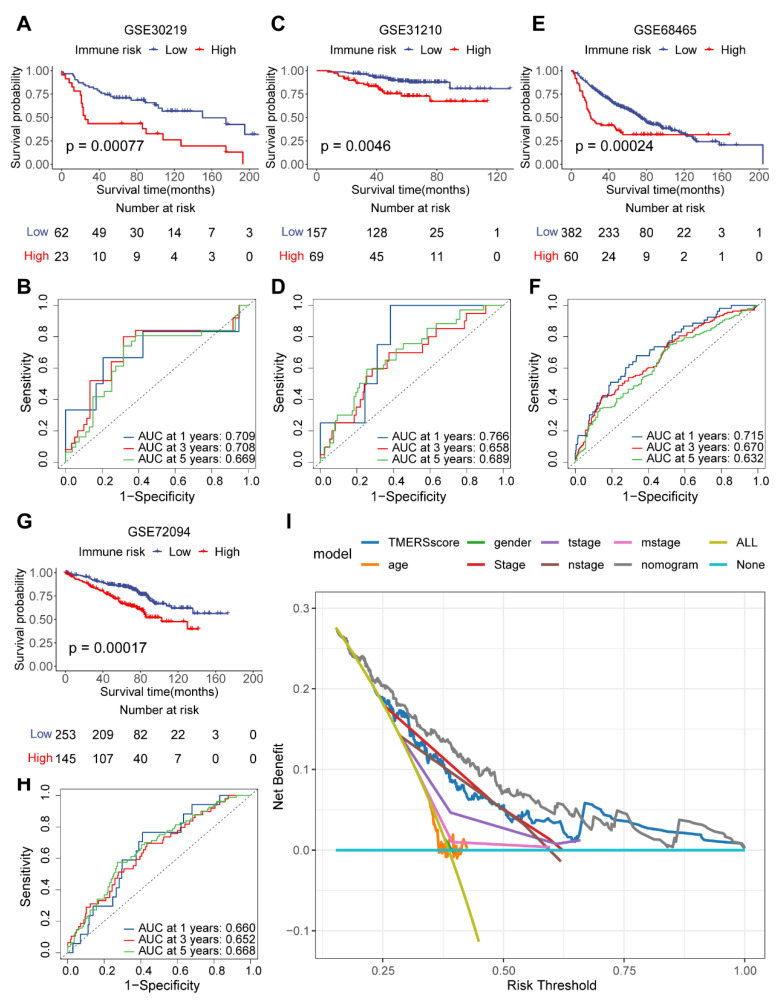
Validation of the performance of TMERSscore to predict survival. (**A**,**C**,**E**,**G**) are the Kaplan-Meier survival curves of TMERSscore in GSE30219, GSE31210, GSE68465, and GSE72094, respectively. (**B**,**D**,**F**,**H**) are the ROC curves of TMERSscore predicting the OS at 1, 3, 5 years in GSE30219, GSE31210, GSE68465, and GSE72094 dataset. (**I**) DCA curves compared the clinical benefit of TMERSscore with other risk factors at 3 years. The none plot represents the assumption that no patients have 3-year survival, while all plots represent the assumption that all patients have 3-year survival at a specific threshold probability. The *x*-axis represents the threshold probabilities, and the *y*-axis measures the net benefit.

We validated the effectiveness of the TMERSscore in prognostic stratification of LUAD patients in four independent datasets containing a total of 1151 LUAD samples, in which all patients were classified into high- and low-risk groups based on the cut-off value obtained from the training cohort. Consistently, OS was significantly worse for patients in the high-risk group than for those in the low-risk group in these validation sets (Figure 3A,C,E,G), with log-rank *p*-values of 0.00077, 0.0046, 0.00024, 0.00017, respectively. The AUCs of the 1, 3, 5-year ROC curves of TMERSscore in GSE30219, GSE31210, and GSE68465 were all around 0.7 (Figure 3B,D,F), and the 1, 3, 5-year AUCs in GSE72094 (Figure 3H) were around 0.65, indicating the robustness and effectiveness of TMERSscore in predicting OS. TMERSscore can distinguish different survival of LUAD patients in the same clinical stage (Supplementary Figure S2).

In addition, we compared the contribution of TMERSscore with other clinical factors, i.e., AJCC pTNM stage, T stage, N stage, age, gender to clinical decision making through DCA curves at 3 years (Figure 3I); the results showed that TMERSscore can provide more benefits to clinical decision making compared to other factors. TMERSscore showed the highest C-index and lowest *p*-value compared to three other published models used to predict the prognosis of LUAD patients (Table 2), which implies that TMERSscore offered the best predictive power.

Overall, TMERSscore showed good predictive performance in the external validation set and in comparison with other prognostic models, with additional benefits for clinical decision making over other clinical risk factors, which implies that TMERSscore can be used to robustly predict the prognosis of LUAD patients.

### 3.3. Revelation of the Underlying Reasons behind Tumor Microenvironment-Related Signature Score

In order to gain biological understanding for the ability of the TMERSscore to stratify and predict survival for LUAD patients, we extracted a total of 223 unique genes contained in the eight selected TMERSs. Next, we obtained the results of differential expression analysis by Limma for 526 tumor samples versus 59 normal lung tissues and high-risk versus low-risk groups in the TCGA cohort; 8136 and 1261 DEGs were identified using |log fold change| > 1 and *p*-value < 0.05 as the screening criteria for DEGs, respectively. The intersections of the two DEG groups totaled 905 genes, and 34 of the 223 genes extracted above were in this intersection, which are thought to contribute more in differential TMERSscore evaluations.

**Figure 4:**
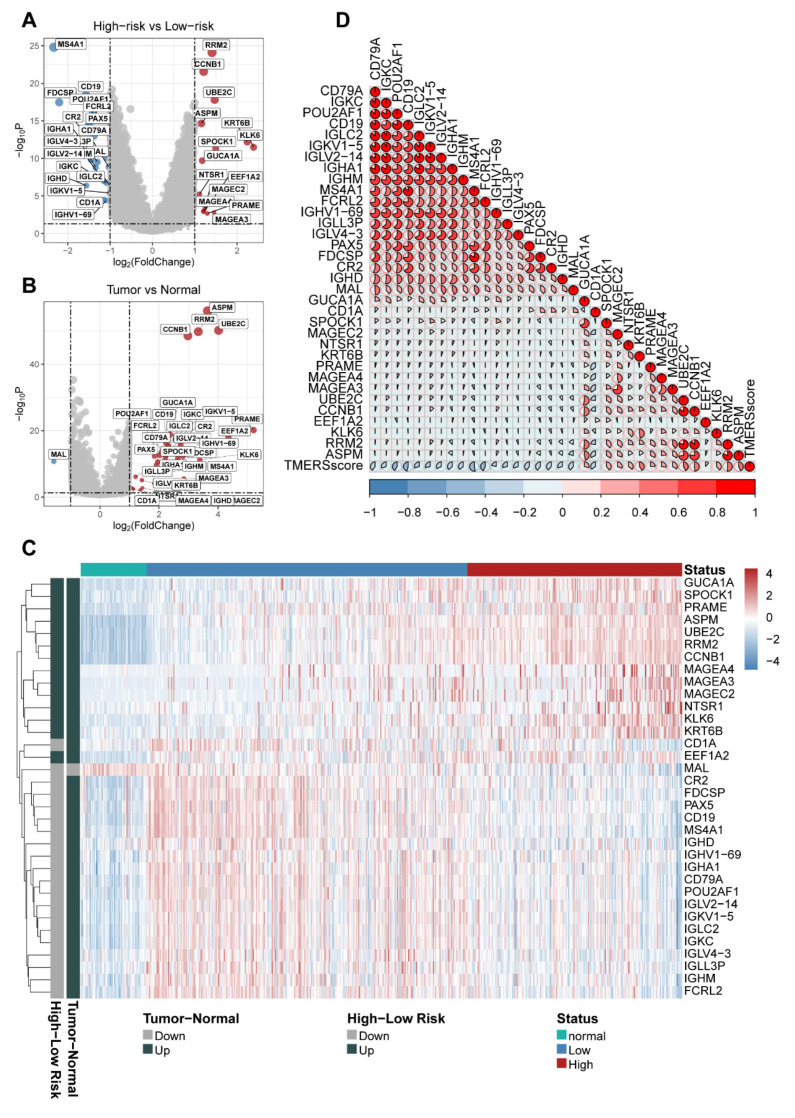
Expression patterns of DEGs contained in the eight selected TMERSs in different subgroups. (**A**,**B**) are volcano plots showing the differential expression status of DEGs in tumors compared to normal and in the high-risk group compared to the low-risk group, respectively. (**C**) Heatmap of the expression of DEGs between different subgroups. (**D**) Correlation plot showing the correlation between TMERSscore and the expression of DEGs.

By comparison, we found that these 31 genes were higher expressed in tumor tissues compared to normal tissues, while *FCRL2*, *IGHM*, *IGLL3P*, *IGLV4-3*, *IGKC*, *IGLC2*, *IGKV1-5*, *IGLV2-14*, *POU2AF1*, *CD79A*, *IGHA1*, *IGHV1-69*, *IGHD*, *MS4A1*, *CD19*, *PAX5*, *FDCSP*, *CR2*, *MAL*, *CD1A* were relatively lower expressed and *ASPM*, *CCNB1*, *EEF1A2*, *KLK6*, *KRT6B*, *MAGEA3*, *MAGEA4*, *MAGEC2*, *NTSR1*, *PRAME*, *RRM2*, *SPOCK1*, *UBE2C* were relatively higher expressed in the high-risk group compared to the low-risk group (Figure 4A–C). Figure 4D shows the correlation between the TMERSscore and the expression level of these genes.

In fact, most of these genes have been reported to have the potential to be prognostic markers or therapeutic targets for patients with LUAD. To give some examples, high levels of *CCNB1* are associated with a poor prognosis in NSCLC [43], and in the early stages of cancer, high levels of *CCNB1* protein are also recognized by the immune system, leading to the production of antibodies and T cells [44]. *RRM2* could independently predicts prognosis in LUAD and the close association of *RRM2* with B-cell marker genes is a potential center of immune response and a key factor in prognosis [45]. *ASPM* expression levels were significantly elevated in lung cancer tissues and were closely associated with LUAD progression [46]. *UBE2C* is a robust prognostic factor for LUAD [47]. *CD19* is present on all B cells and is a reliable biomarker for B lymphocyte development and lymphoma diagnosis, as well as a target for leukemia immunotherapy [48]. The *CD79A*/*CD40* co-stimulatory structural domain confers enhanced proliferative capacity of CAR-T cells and increased anti-tumor effects in mouse models [49]. *MS4A1* encodes the *CD20* protein [50], which is the target of active drugs for the treatment of autoimmune diseases such as rituximab, ocrelizumab, obinutuzumab, ofatumumab, ibritumomab tiuxetan, tositumomab, and ublituximab. The OCA-B protein encoded by *POU2AF1* promotes the expression of T-cell target genes in the presence of repeated antigen exposure [51]. These explain the validity and reliability of the TMERSscore in predicting the prognosis of LUAD patients and in assessing the response to immunotherapy. Furthermore, some of these genes may become key targets for future LUAD treatment or combination therapy targeting several of these genes may play an important role in the future treatment of LUAD.

**Figure 5:**
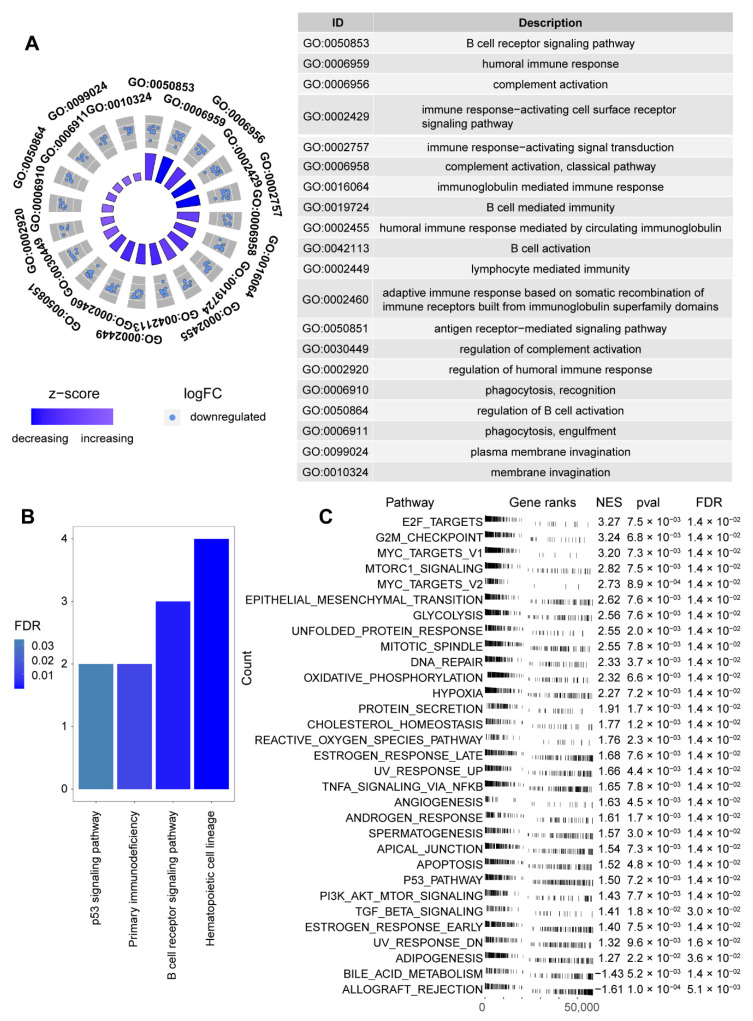
Enrichment analysis results relevant to TMERSscore. (**A**) Gene ontology analyses of the DEGs. (**B**) KEGG analyses of the pathways of the DEGs. (**C**) GSEA of TMERSscore demonstrates the activation pathways of different subgroups.

To further elucidate the potential biological functions and pathways associated with TMERSscore, we performed GO enrichment and KEGG enrichment analysis based on the 34 DEGs. The top 20 GO terms presented in Figure 5A show a significant enrichment of DEGs in B cell-related as well as immune response-related terms such as “B cell receptor signaling pathway”, “B cell activation”, “humoral immune response”, “immune response-activating cell surface receptor signaling pathway”, and “lymphocyte mediated immunity”, among others. The results of KEGG enrichment analysis in Figure 5B show that the DEGs were mainly enriched in pathways such as “Hematopoietic cell lineage”, “B cell receptor signaling pathway”, “Primary immunodeficiency”, and “p53 signaling pathway”. In addition, Gene Set Enrichment Analysis (GSEA) was used to demonstrate differential activated pathways between high and low risk groups (Figure 5C), which showed that pathways important for LUAD development and immunology, such as E2F_TARGETS, G2M_CHECKPOINT, MYC_TARGETS, GLYCOLYSIS, UNFOLDED_PROTEIN_RESPONSE, DNA_REPAIR, HYPOXIA were significantly activated in the high-risk group compared to the low-risk group, consistent with the high-risk characteristics.

### 3.4. Evaluation of Tumor Malignancy and ICI Immunotherapy Efficacy by Tumor Microenvironment-Related Signature Score

**Figure 6:**
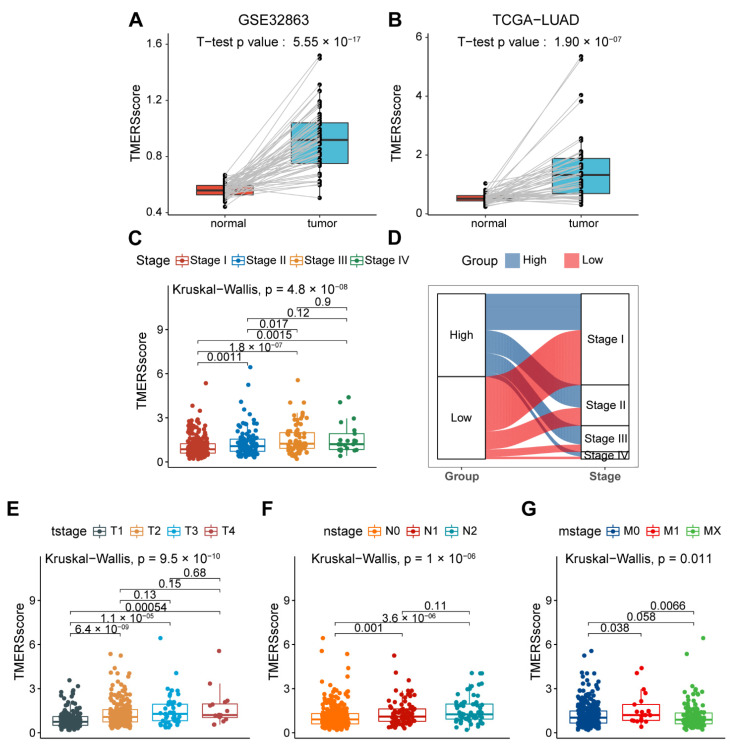
Evaluation of tumor malignancy by TMERSscore. (**A**) Pairwise boxplot of paired normal and tumor samples in the GSE32863 dataset. (**B**) Pairwise boxplot of paired normal and tumor samples in the TCGA cohort. (**C**,**E**–**G**) Boxplots showing the difference between the TMERSscore at different AJCC pTNM Stage, T stage, N stage, and M stage, respectively. (**D**) Sankey diagram showing the relationship between high and low TMERSscore subgroups and AJCC Stages.

In order to determine whether the evaluation of TMERSscore is consistent with other risk stratification methods, we compared the TMERSscores among different subgroups in several datasets. Firstly, in both the GSE32863 cohort and the TCGA cohort, the TMERSscores of the tumors were significantly higher than paired normal lung tissue samples, indicating that the TMERSscore was significantly increased when normal lung tissues progressed to tumors (Figure 6A,B).

Secondly, as the American Joint Committee on Cancer (AJCC) TNM stage is now the standard staging method for NSCLC, and the AJCC pTNM stage is a comprehensive assessment of the TNM stage, we compared the TMERSscores at different clinical stages. As showed in Figure 6C, the TMERSscore of patients in early stage (Stage I and Stage II) was significantly lower than that of patients in advanced stage (Stage III and Stage IV), and there was an increasing trend of TMERSscore with tumor progression, which could also be observed in different stages of T, N, and M (Figure 6E–G). Sample distributions were explored through a Sankey diagram depicting the Stage–TMERSscore relationships where most early-stage (Stage I and Stage II) patients had low TMERSscore while most late-stage (Stage III and Stage IV) patients had high TMERSscore (Figure 6D). It was also observed that a proportion of early-stage patients were considered high risk and a proportion of late stage patients were considered low risk, suggesting that the TMERSscore may provide some complementary and incremental predictive value to the existing staging system. In fact, some studies have reported differences in recurrence and survival among LUAD patients in each AJCC staging subgroup [52].

**Figure 7:**
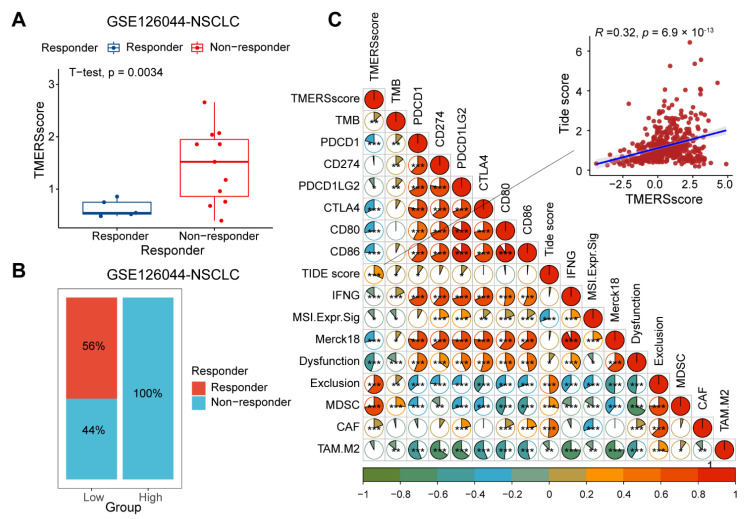
Evaluation of ICI therapeutic efficacy of LUAD patients by TMERSscore. (**A**) Boxplot demonstrating the difference in TMERSscore between responder and non-responder LUAD patients in the GSE126044 dataset receiving anti-PD-1 treatment. (**B**) Bar graph illustrating the percentage of clinical response to anti-PD-1 immunotherapy in the high and low TMERSscore groups for the GSE126044 dataset. (**C**) Correlations between TMERSscore values, TMB, PD-1, PD-L1, CTLA-4, CD80, CD86, TIDE score, IFNG, MSI.Expr.Sig, Merck18, Dysfunction, Exclusion, MDSC, CAF, TAM.M2 scores in the TCGA cohort. Different correlations between two signatures are indicated by different colors. * *p* < 0.05, ** *p* < 0.01, *** *p* < 0.001.

Furthermore, we explored whether TMERSscore could provide an effective instruction for immunotherapy. Here, gene expression profiles of NSCLC patients treated with anti-PD-1 therapy in GSE126044 and SKCM patients treated with ICI therapy obtained from CRI iAltas were used to evaluate whether TMERSscore could be used as a marker to assess the efficacy of ICI treatment. As a result, we found that the TMERSscore of responding patients in GSE126044 was significantly lower than that of non-responding patients (Figure 7A), and that patients belonging to the low-risk group had a better response to ICI therapy compared to patients in the high-risk group according to the cut-off values obtained from TCGA cohort (Figure 7B).

TMB and TIDE score are recognized as effective methods to predict immunotherapy response in patients with solid tumors, so we obtained the TMB of patients and calculated the corresponding TIDE score results based on the gene expression files of LUAD patients in the TCGA cohort. The correlation between markers is shown in Figure 7C, from which it can be found that TMERSscore is weakly positively correlated with TMB because TMB reflects the immunogenicity of the tumor and predicts improved survival after immunotherapy; however, in cancer patients not receiving immunotherapy, high TMB tends to be associated with poor prognosis, which may be due to the accumulation of mutations during tumor progression due to genomic instability, implying that high TMB is usually associated with advanced tumors. TMERSscore had a strong positive correlation with TIDE score, implying that a low TMERSscore characterizes a better immunotherapy response as well as a low TIDE score. In addition, it was found that TMERSscore was highly negatively correlated with T-cell dysfunction score and T-cell exclusion score, and was highly positively correlated with the scores of reported cell types that suppress tumor T-cell infiltration, such as cancer-associated fibroblasts (CAFs) and myeloid-derived suppressor cells (MDSCs), but weakly correlated with the scores of tumor-associated macrophage M2 (TAM.M2) subtypes, implying that high TMERSscore may predict an immunosuppressive environment, while low TMERSscore predicts a tumor microenvironment that favors immunotherapeutic response.

**Figure 8:**
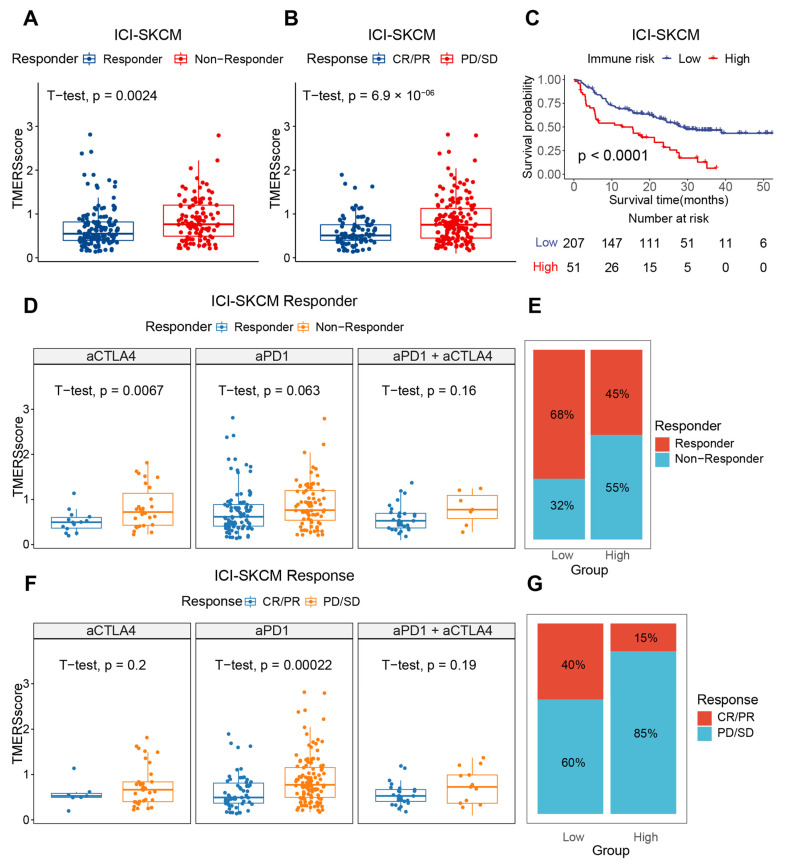
Evaluation of ICI therapeutic efficacy by TMERSscore in SKCM cohort. (**A**,**B**) Boxplots showing the difference in TMERSscore between responders and non-responders, and between CR/PR subgroups and PD/SD subgroups in the SKCM cohort receiving ICI treatment, respectively. (**C**) Kaplan-Meier survival curve of the TMERSscore in ICI-treated SKCM cohort. (**D**,**F**) Boxplots showing the difference between responders and non-responders of TMERSscore in the SKCM cohort for different ICI therapies, i.e., anti-CTLA4, anti-PD-1, anti-PD-1 combined anti-CTLA4, and between the CR/PR subgroup and the PD/SD subgroup, respectively. (**E**,**G**) Bar charts showing the percentage of responders vs. non-responders and CR/PR vs. PD/SD in different TMERSscore subgroups in the SKCM cohort, respectively. Responders include CR, PR, and PD response subgroups, and non-responders include SD response subgroups.

In the ICI-treated SKCM cohort, we compared the differences in TMERSscore among different immunotherapy response subgroups. Consistently, the TMERSscore of non-responders was significantly higher than that of responders (Figure 8A), where most of the high TMERSscore in responders belonged to the PD subgroup (Figure 8B), implying that TMERSscore can reflect specific immunotherapy response to some extent. The TMERSscore had different predictive performance for different ICIs and had greater discriminatory power for individual ICIs than for multiple immune checkpoint combinations (Figure 8D,F). In addition, the classification of high and low risk groups revealed significant differences in the OS of patients (Figure 8C), and more patients in the low-risk group had a good response to immunotherapy than in the high-risk group (Figure 8E,G).

## 4. Discussion

A number of studies have emerged showing the effectiveness of assessing patient survival and treatment efficacy from the TME [53,54,55,56]. In the present study, we constructed a TME-based scoring system for LUAD patient stratification and validated it in several independent external datasets. Our study found that TMERSscore is an independent predictor of LUAD patients, which not only significantly stratifies LUAD patients in terms of prognosis, but also positively correlates with TIDE score, positively correlates with tumor malignancy, and is significantly lower in responders than in non-responders in the dataset receiving ICI immunotherapy. This implies that the TMERSscore can also stratify the treatment response of LUAD patients to evaluate the benefit from immunotherapy. Overall, the TMERSscore may provide independent and incremental predictive value for existing markers.

Based on the collected TME-related gene sets, it is convenient to obtain the bulk gene expression of LUAD patients and calculate the quantitative enrichment results of ssGSEA for each gene set that represents different properties of TME, such as infiltrating immune cells or corresponding TME characteristics as defined in the literature. Therefore, we constructed a predictive model based on eight significant TMERSs selected by LASSO-Cox analysis from the quantitative results of these gene sets. Signatures encompassed in the model such as T cells, B cell lineages, and MDSCs proved to be significant in relation to tumorigenesis and progression as well as immunotherapy response. We also extracted 34 genes contained in these eight selected TMERSs with differential abundance in both tumor versus normal and high versus low TMERSscore, most of which were reported to be independent prognostic factors for LUAD (e.g., *CCNB1*, *RRM2*) or excellent therapeutic targets for leukemia and some autoimmune diseases (e.g., *CD19*, *MS4A1*). This reveals the reliability of TMERSscore to predict survival and immunotherapy response in LUAD patients. In addition, these genes were found to be enriched in B cell-related as well as immune response-related GO and KEGG terms (e.g., B cell receptor signaling pathway, humoral immune response, primary immunodeficiency), and GSEA analysis showed that many important pathways are activated in high TMERSscore subtypes (e.g., E2F_TARGETS, G2M_CHECKPOINT). Although the results of the enrichment analysis showed that the TMERSscore established in this study tended to exhibit a B cell-related signature, we believe that it should be a comprehensive assessment of the tumor microenvironment, in which B cell-related predictors play a more relevant role,. Recent studies have shown that B cell-related signatures are effective predictors of ICI immunotherapy benefit in lung adenocarcinoma patients [57]. Furthermore, according to some recent studies in clinical cohorts, B cells have been found to play an important role in the immune response. After antigen exposure, B cells can be broadly subdivided into B cells involved in antibody-mediated immune responses and B cells that regulate immune responses, similar to the concept of regulatory T cells. These B regulatory cells characterized by *IL10* production (Breg) [58] have been shown to interfere with the immune response in several diseases, including cancer. There are also data suggesting that B cells have antigen-presenting and proinflammatory effects [58,59,60]. This is consistent with a recent report by Petitprez and his co-authors [61] that B cells located partially in the tertiary lymphoid structures were strong positive predictors of ICI response in sarcoma patients receiving pembrolizumab in a phase 2 clinical trial. These suggest that B cell-associated predictors can play a role in play an important role in the prediction of response to immunotherapy.

TMERSscore exhibited robust performance in predicting prognosis, as it performed well in four independent external validation sets. The Kaplan-Meier survival curves verified the ability of TMERSscore to stratify prognosis, the ROC curves confirmed the good sensitivity and specificity of TMERSscore in predicting OS, and the DCA curves showed the greater contribution of TMERSscore to clinical decision making compared to other clinical risk factors. TMERSscore also showed the highest C-Index and the lowest *p*-value compared to three published models for predicting OS in LUAD patients. Furthermore, TMERSscore was positively correlated with disease progression and was able to predict survival outcomes for patients in different TNM stages.

Immunotherapy using ICIs is a revolutionary treatment that can be applied to multiple cancer types, including LUAD. Although ICI therapy improves clinical outcomes in patients with solid tumors, only a small subset of patients responds; thus, the discovery of markers that can predict the response to immunotherapy in patients is of great importance and remains a great challenge. It has been shown that immune checkpoint expression levels, MSI, and TMB are not consistently predictive of assessing the benefit of immunotherapy for patients [62,63,64], and a growing number of studies suggest that different TME phenotypes may represent different survival outcomes as well as different responses to immunotherapy [65]. Our results showed that TMERSscore assessed survival and response to immunotherapy from the perspective of TME-related signatures, and among patients receiving immunotherapy, TMERSscore was significantly lower in responders than in non-responders. We also compared the TMERSscore with a series of results (including T-cell dysfunction score and T-cell exclusion score) calculated from the TIDE web application. The correlation between the signatures shows that the TMERSscore is highly positively correlated with the T-cell exclusion score, and from the MDSC score given by TIDE and the MDSC features contained within the TMERSscore, we can speculate that MDSCs may play a relatively more important role in T-cell exclusion and that therapies targeting MDSCs may help improve immunotherapy resistance in LUAD patients. Further, a strong negative correlation was found between T-cell exclusion scores and levels of tumor cytotoxic T lymphocytes, while higher cytotoxic T lymphocytes levels in metastatic melanoma indicate higher patient survival rates [66], which is consistent with a low TMERSscore predicting better survival. TMERSscore also showed a strong negative correlation with T-cell dysfunction scores, implying that T cells in the TME with low TMERSscore may have early dysfunction and that anti-PD-1 therapy can revive early dysfunctional T cells [67], implying that low TMERSscore predicts more benefit from ICI therapies. Supportive results can be found in the evaluation of response of patients receiving immunotherapy by TMERSscore.

Therefore, TMERSscore, to some extent, represents the heterogeneity of the TME and may be used as a biomarker to evaluate the prognosis and the response to ICI immunotherapy in LUAD patients. B-cell related features are also included within the model and some genes that make more contributions are more enriched in B-cell related GO and KEGG terms; however, current approaches to assess the benefit of immunotherapy are mostly from a T-cell perspective, so the TMERSscore may provide new insights into assessing the benefit of immunotherapy from a comprehensive perspective that includes B cells, thus providing independent and incremental predictive value over other biomarkers.

There are some limitations in this study. First we listed 303 TME-related signatures but only eight of them were used. The application of the remaining signatures in prognosis and immunotherapy response assessment needs to be further explored. In addition, limited by the open source LUAD dataset with immunotherapy recipients, we did not complete our validation in a large dataset, and the immunotherapy response prediction-related validation was done in the SKCM dataset and by comparing to the TIDE scores. Therefore, further validation needs to be completed in a larger LUAD dataset with immunotherapy information to exclude differences between cancer types for more reliable results.

## 5. Conclusions

Overall, we constructed a TME-based scoring system that can be used for LUAD patient stratification as well as treatment efficacy assessment. Supportive results from external validation sets have shown its robustness and effectiveness as a potential biomarker for assessing prognosis and treatment efficacy.

## Figures and Tables

**Table 1:** Clinical characteristics of LUAD patients included in the construction and validation of the prognostic model.

Characteristics	Cohort				
	Train	Validation			
	TCGA	GSE30219	GSE30210	GSE68465	GSE72094
ALL	479	85	226	442	398
Age, average (standard deviation)	65.2 (10.1)	61.5 (9.28)	59.6 (7.40)	64.4 (10.1)	69.4 (9.45)
Gender:					
female, %	254 (53.0%)	19 (22.4%)	121 (53.5%)	219 (49.5%)	222 (55.8%)
male, %	225 (47.0%)	66 (77.6%)	105 (46.5%)	223 (50.5%)	176 (44.2%)
AJCC pTNM Stage:					
I, %	264 (55.1%)	not reported	168 (74.3%)	not reported	254 (63.8%)
II, %	118 (24.6%)	not reported	58 (25.7%)	not reported	67 (16.8%)
III, %	76 (15.9%)	not reported	0 (0%)	not reported	57 (14.3%)
IV, %	21 (4.38%)	not reported	0 (0%)	not reported	15 (3.77%)
unknown	0 (0%)	not reported	0 (0%)	not reported	5 (1.26%)
M stage:					
M0, %	321 (67.0%)	85 (100%)	not reported	not reported	not reported
M1, %	21 (4.38%)	0 (0%)	not reported	not reported	not reported
MX, %	137 (28.6%)	0 (0%)	not reported	not reported	not reported
T stage:					
T1, %	163 (34.0%)	71 (83.5%)	not reported	150 (33.9%)	not reported
T2, %	255 (53.2%)	12 (14.1%)	not reported	251 (56.8%)	not reported
T3, %	45 (9.39%)	2 (2.35%)	not reported	28 (6.33%)	not reported
T4, %	16 (3.34%)	0 (0%)	not reported	11 (2.49%)	not reported
unknown	0 (0%)	0 (0%)	not reported	2 (0.45%)	not reported
N stage:					
N0, %	322 (67.2%)	82 (96.5%)	not reported	299 (67.6%)	not reported
N1, %	90 (18.8%)	3 (3.53%)	not reported	87 (19.7%)	not reported
N2, %	67 (14.0%)	0 (0%)	not reported	53 (12.0%)	not reported
NX, %	0 (0%)	0 (0%)	not reported	3 (0.68%)	not reported

**Table 2:** Comparison of C-index and *p*-value of different models in TCGA-LUAD cohort.

Signatures	C-Index	*p*-Value
TMERSscore	0.6719824	3.231601 × 10^−13^
Lei_Liu_2021	0.6719603	1.812015 × 10^−12^
Lulu_He_2020	0.6653507	8.032247 × 10^−13^
Weishuang_Ma_2021	0.6617354	2.155125 × 10^−11^
age	0.5110899	0.6784349
gender	0.5315841	0.1449488
AJCC pTNM Stage	0.6437586	1.533862 × 10^−13^

## Data Availability

The datasets in this study can be found online. The names and login numbers of the repositories can be found in the article/Supplementary Materials.

## Supplementary Materials

The following supporting information can be downloaded at: https://www.mdpi.com/article/10.3390/genes13060951/s1, Table S1: Tumor microenvironment related signatures (n = 303) and their contained genes; Table S2: Gene expression profiles of SKCM patients receiving ICI immunotherapy downloaded from CRI iAltas; Table S3: Metadata of SKCM patients receiving ICI immunotherapy downloaded from CRI iAltas; Table S4: Results of univariate Cox regression analysis of significant tumor microenvironment-related signatures; Figure S1: Nomogram and calibration curve based on TMERSscore built in TCGA-LUAD cohort; Figure S2: Kaplan-Meier survival curves of TMERSscore in different clinical-stage subgroups.
