# Supplementary material for: Construction and Validation of a Tumor Microenvironment-Based Scoring System to Evaluate Prognosis and Response to Immune Checkpoint Inhibitor Therapy in Lung Adenocarcinoma Patients
Source: Genes (Basel). 2022 May 26;13(6):951. doi: 10.3390/genes13060951 (PMC9222903; doi:10.3390/genes13060951)
Supplement: Supplementary file 1 [file genes-13-00951-s001.zip › genes-1721155-Supplementary_Material.pdf]

Supplementary Material

1 Supplementary Figures and Tables

1.1 Supplementary Figures

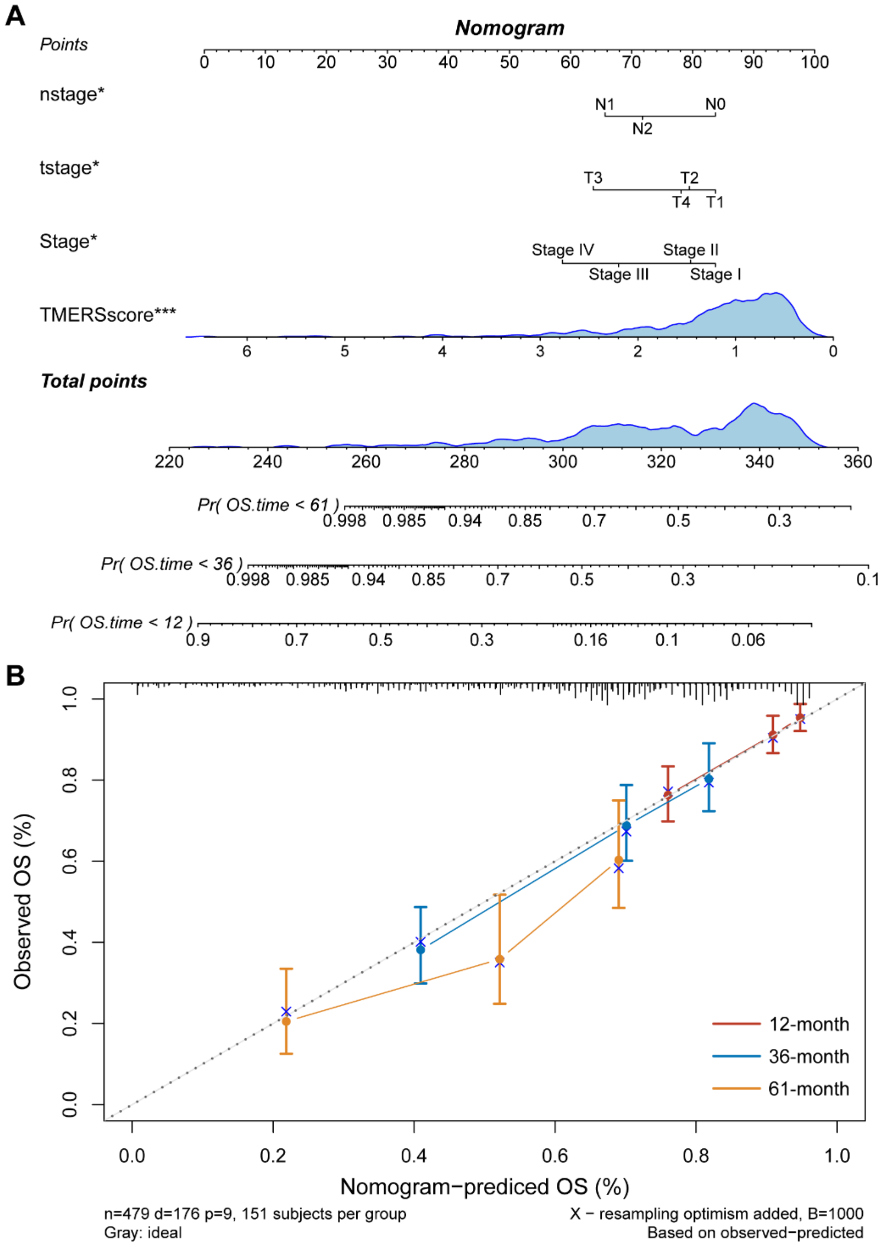

Supplementary Figure S1. Nomogram and calibration curve based on TMERSscore built in TCGA-LUAD cohort. (A) Nomogram for predicting overall survival at 1, 3, and 5 years. (B) Calibration curves for 1, 3 and 5 year overall survival in LUAD patients. * p < 0.05, *** p < 0.001.

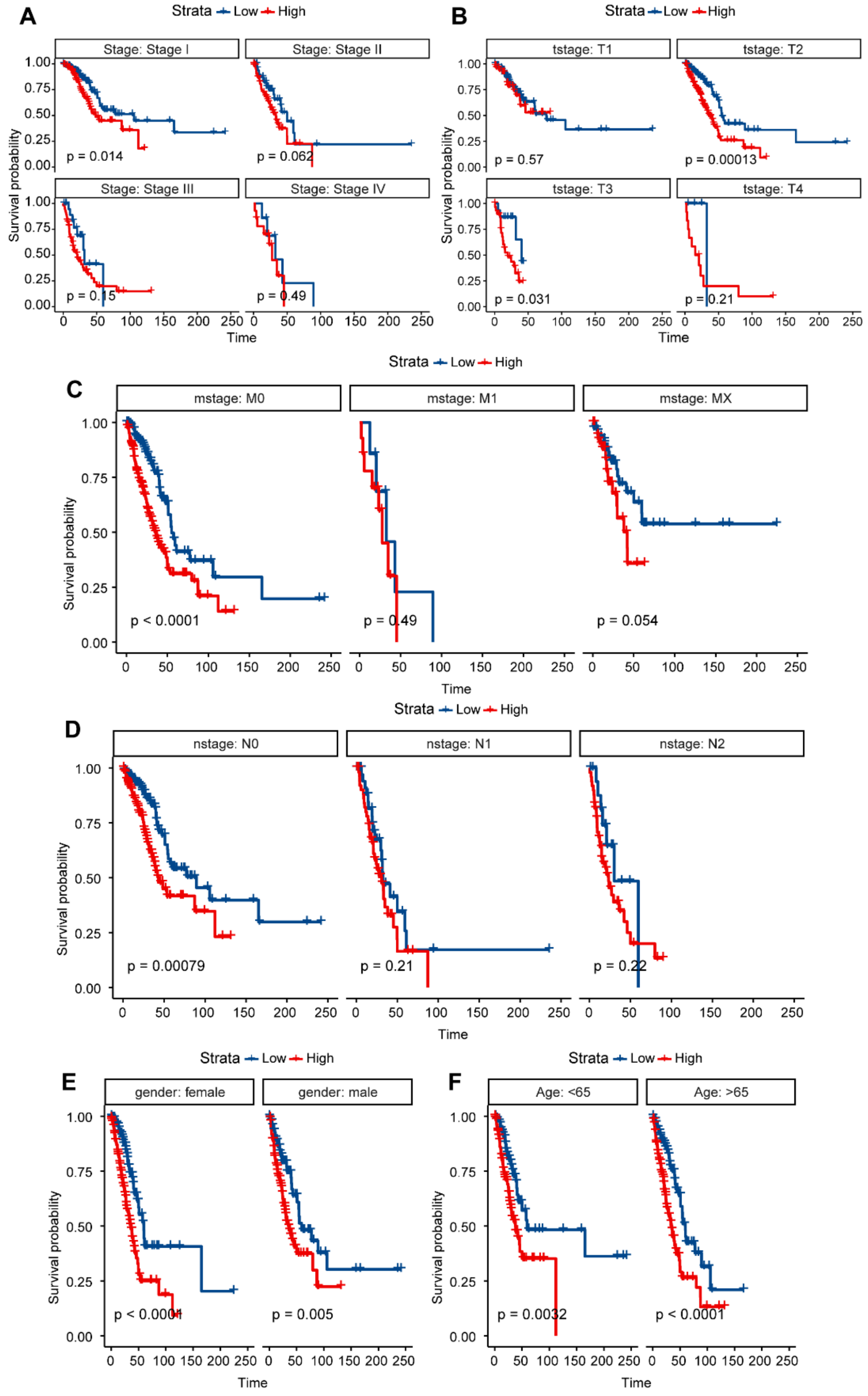

Supplementary Figure S2. Kaplan-Meier survival curves of TMERSscore in different clinical-stage subgroups. (A) (B) (C) (D) (E) (F) are the survival curves of TMERSscore in different AJCC pTNM stage, T stage, M stage, N stage, gender, and age subgroups, respectively.

1.2 Supplementary Tables S1-S4

Supplementary Table S1. Tumor microenvironment related signatures (n = 303) and their contained genes.

Supplementary Table S2. Gene expression profiles of SKCM patients receiving ICI immunotherapy downloaded from CRI iAltas.

Supplementary Table S3. Metadata of SKCM patients receiving ICI immunotherapy downloaded from CRI iAltas.

Supplementary Table S4. Results of univariate Cox regression analysis of significant tumor microenvironment-related signatures.
