# Supplementary figures and images for: Construction and Validation of a Tumor Microenvironment-Based Scoring System to Evaluate Prognosis and Response to Immune Checkpoint Inhibitor Therapy in Lung Adenocarcinoma Patients
Source: Genes (Basel). 2022 May 26;13(6):951. doi: 10.3390/genes13060951 (PMC9222903; doi:10.3390/genes13060951)

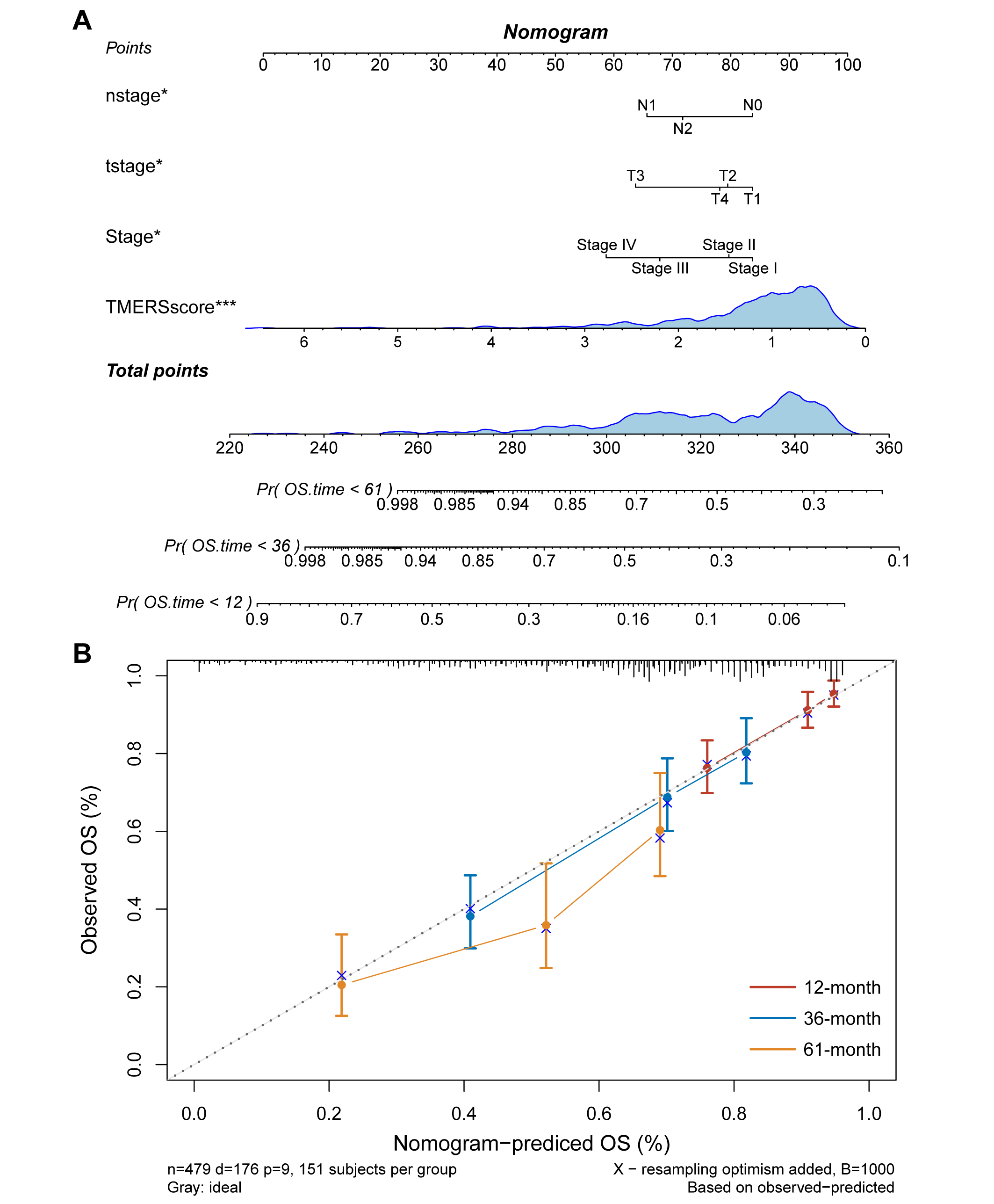

Supplement: Supplementary file 1 [file genes-13-00951-s001.zip › Supplementary figure S1.tif]

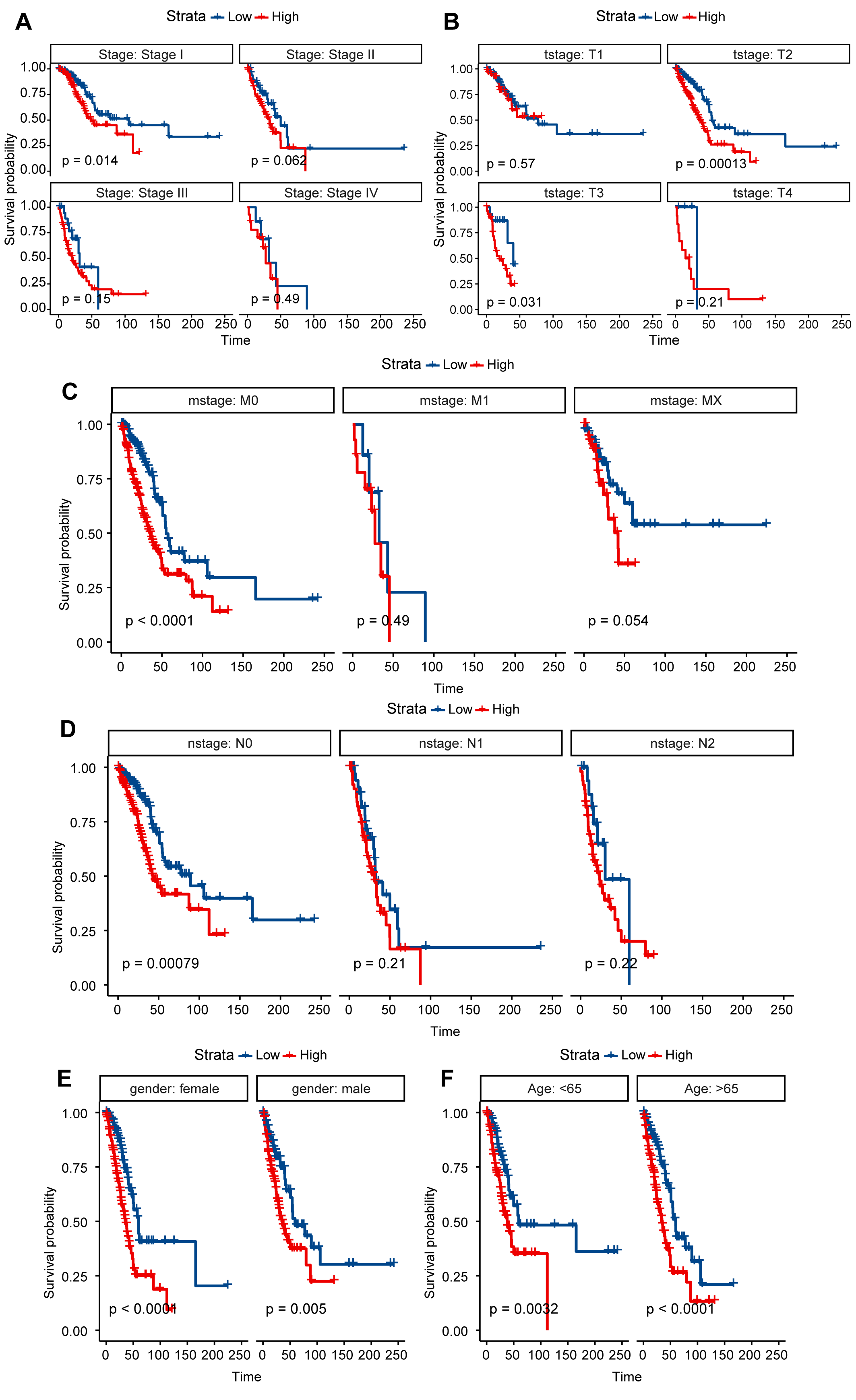

Supplement: Supplementary file 1 [file genes-13-00951-s001.zip › Supplementary figure S2.tif]
